# PES inhibits human-inducible Hsp70 by covalent targeting of cysteine residues in the substrate-binding domain

**DOI:** 10.1074/jbc.RA120.015440

**Published:** 2020-12-24

**Authors:** Jie Yang, Weibin Gong, Si Wu, Hong Zhang, Sarah Perrett

**Affiliations:** 1 National Laboratory of Biomacromolecules, CAS Center for Excellence in Biomacromolecules, Institute of Biophysics, Chinese Academy of Sciences, Beijing, China; 2 University of the Chinese Academy of Sciences, Beijing, China

**Keywords:** Hsp70, PES, covalent inhibitor, cysteine modification, BCA, bicinchoninic acid, CSM, center of spectral mass, FAR, FITC-labeled ALLLSAPRR peptide, FP, fluorescence polarization, hHsc70, human HspA8, hHsp70, human HspA1A, HSQC, heteronuclear single quantum coherence, ITC, isothermal titration calorimetry, MLKL, mixed lineage kinase domain-like protein, mP, millipolarization units, NBC1, necroptosis-blocking compound 1, NBD, nucleotide-binding domain, NEF, nucleotide exchange factor, PES, 2-phenylethynesulfonamide or pifithrin-μ, ROS, reactive oxygen species, RT, room temperature, SBD, substrate-binding domain, SBDα, C-terminal α-helical lid subdomain of SBD, SBDβ, β-sheet substrate-binding subdomain, SEC, size-exclusion chromatography

## Abstract

Hsp70 proteins are a family of ancient and conserved chaperones. They play important roles in vital cellular processes, such as protein quality control and the stress response. Hsp70 proteins are a potential drug target for treatment of disease, particularly cancer. PES (2-phenylethynesulfonamide or pifithrin-μ) has been reported to be an inhibitor of Hsp70. However, the mechanism of PES inhibition is still unclear. In this study we found that PES can undergo a Michael addition reaction with Cys-574 and Cys-603 in the SBDα of human HspA1A (hHsp70), resulting in covalent attachment of a PES molecule to each Cys residue. We previously showed that glutathionylation of Cys-574 and Cys-603 affects the structure and function of hHsp70. In this study, PES modification showed similar structural and functional effects on hHsp70 to glutathionylation. Further, we found that susceptibility to PES modification is influenced by changes in the conformational dynamics of the SBDα, such as are induced by interaction with adjacent domains, allosteric changes, and mutations. This study provides new avenues for development of covalent inhibitors of hHsp70.

Hsp70 is the central hub for the protein quality control machinery. Hsp70 proteins participate in protein folding and refolding, help prevent protein aggregation, and facilitate protein assembly, disassembly, degradation, and the stress response (1). Hsp70 can interact with numerous protein clients and is involved in diverse physiological activities, including signal transduction, apoptosis, and transmembrane transport (2, 3, 4). Therefore, Hsp70 proteins are also related to some disease processes, including neurodegeneration, infection, and many types of cancer (3, 5, 6).

Hsp70 proteins contain two individual domains, namely the ATPase or nucleotide-binding domain (NBD) and the substrate-binding domain (SBD), connected by a flexible linker (7). The SBD can be further divided into a β-sheet substrate-binding subdomain (SBDβ) and a C-terminal α-helical lid subdomain (SBDα) (8). Allosteric conformational changes in Hsp70 couple the ATP hydrolysis cycle in the NBD and the substrate binding/release cycle in the SBD (9). In the ATP-bound state, the NBD and SBD of Hsp70 dock into a single entity, and the SBDα detaches from the SBDβ and leans against the NBD, resulting in low substrate affinity (10, 11). Substrate binding to the SBDβ promotes undocking between the NBD and SBD of the *E. coli* homolog of Hsp70, Dnak (12), but has a weaker effect on undocking of human cytoplasmic Hsp70 (13). In the ADP-bound state, the NBD and SBD tend to undock, separating the two individual domains, and the SBDα covers the SBDβ like a lid, resulting in high substrate affinity, although a significant proportion of ADP-bound human cytoplasmic Hsp70 remains docked compared with the largely undocked conformation of ADP-bound DnaK (7, 12, 13, 14). Cochaperones such as Hsp40 and nucleotide exchange factors (NEFs) can affect nucleotide hydrolysis/exchange and substrate binding/release to regulate the functional cycle of Hsp70 (9).

Different Hsp70 family members perform specific and overlapping functions. For *Homo sapiens* at least 13 typical Hsp70 members have been identified in cells. Among them HspA1A is the cytosolic stress-induced form (human HspA1A [hHsp70]) and HspA8 is the cytosolic constitutively expressed form (human HspA8 [hHsc70]) (15). If both *HSPA1A* and *HSPA8* genes are silenced by siRNA, the survival rate of cells is very low (16). Expression of hHsp70 is very low under normal conditions but rises dramatically under stress conditions and in some cancer cells (17). In addition to its fundamental function in protein quality control, hHsp70 also plays antistress and antisenescence roles (17). Expression of hHsc70 is abundant and stable (18). Besides the overlap of fundamental functions with hHsp70, hHsc70 plays a role in multiple cellular processes, such as clathrin coat disassembly and chaperone-mediated autophagy (18).

Heat shock proteins play critical roles in rapid cell division, metastasis, and evasion of apoptosis of cancer cells through their function in protein quality control (19). Hsp90 is responsible for the final maturation of about 200 client proteins, including some oncogene products (20). Hsp90 has been successfully linked to cancer therapy (20). Inhibitor development and clinical trials of Hsp90 have continued to expand rapidly. However, drug resistance of Hsp90 inhibitors has forced people to consider Hsp70 alone or combined with Hsp90 as a target for cancer therapy, since Hsp70 often cooperates with Hsp90 in protein maturation and is also responsible for ultimate maturation of some proteins (21). Although the number of Hsp70 inhibitors is growing rapidly with the development of plate screening, there is still no breakthrough in terms of clinical trials. Inhibitors of Hsp70 can be divided into three general types according to their binding site and mechanism. The first type targets the NBD of Hsp70. The candidates include 15-DSG, MKT-077, VER-155008, and YK5, which generally interfere with nucleotide binding to the NBD (16, 22, 23). The second type targets the SBD of Hsp70. The candidates include PES, PES-Cl, PET-16, novolactone, and ADD70, which generally affect substrate binding to the SBD or allostery of Hsp70 (22, 24, 25, 26, 27). The third type targets the interaction interface of Hsp70 and cochaperones, which then disrupts cooperation between Hsp70 and cochaperones. The candidates include MAL3-101, myricetin, and YM-1 as well as their derivatives (28, 29, 30).

Among these Hsp70 inhibitors, PES has attracted intensive study. It was first identified as a p53 inhibitor (31). In 2009, Donna L. George’s lab identified PES as an Hsp70 inhibitor and suggested that its inhibition is related to the SBDα of hHsp70 (24). They also found that PES interacts more strongly with hHsp70 than hHsc70, and PES is more toxic to tumor cells than normal cells (24). PES-Cl, a derivative of PES, is more efficient at killing cancer cells than PES (25). Efforts are underway to develop PES as an anticancer drug, alone or for combination therapy (32, 33, 34, 35). However, the mechanism by which PES and its derivatives inhibit Hsp70 is still an enigma. Although George and coworkers detected interaction between hHsp70 and PES by ITC (36), there is still no high-resolution structure available for the complex of hHsp70 and PES, so detailed information regarding the mode of interaction is still lacking. Some research suggests that PES can act like a detergent (16).

Cytotoxicity of PES was found to be related to elevation of ROS in cells (37). PES was suspected to cause covalent modification and cross-linking of p53 (38). Since PES is an alkyne *i.e.*, it contains a carbon–carbon triple bond, which is adjacent to an electron-withdrawing sulfonamide group, PES is predicted to be a potential Michael acceptor (Fig. 1). The Michael addition reaction of thiols in peptides with compounds containing a carbon–carbon triple bond has been demonstrated previously (39, 40). This suggests that PES and its derivatives could react with thiol groups within proteins. To explore whether PES can react with thiols in Hsp70 proteins, we tested whether PES can covalently attach to hHsp70 and further measured the effect of PES covalent modification on the structure and function of hHsp70. We also probed the factors that affect PES modification of hHsp70 and compared the reactivity of PES with hHsp70 and hHsc70. This study provides an approach for development of covalent inhibitors of hHsp70.

**Figure 1:**
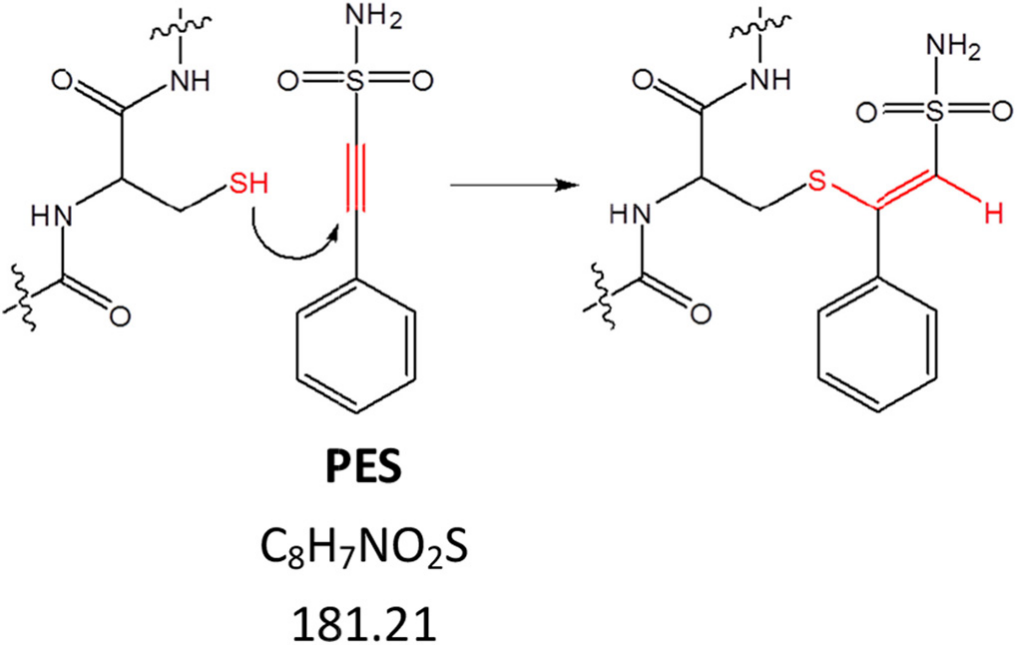
**Schematic diagram of the Michael addition reaction between cysteine residues in hHsp70 and PES**. hHsp70, human HspA1A.

## Results

### Detection of covalent attachment of PES to the SBDα of hHsp70

The stable interaction between PES and hHsp70 was previously demonstrated by pull-down of proteins in tumor cells using biotin-labeled PES, and the interaction was found to be related to the SBDα of hHsp70 (24). However, the mechanism of this interaction remains unknown. In our previous study, we found that Cys-574 and Cys-603 are reactive and readily undergo modification (41). As PES is an alkyne, there is the potential for it to undergo a Michael addition reaction with protein thiol groups (39, 40). To test whether PES can react with hHsp70, we incubated PES and hHsp70 at different concentration ratios and for different time periods in the presence of ADP at 37 °C. Size-exclusion chromatography (SEC) can be used to monitor cysteine modifications of SBDα (41). At low concentration ratios (<10:1) of PES to hHsp70 and short incubation times (<12 h), we just observed a weak peak shift in SEC. When the concentration ratio of PES to hHsp70 reached 100:1 and the incubation time reached 24 h, an obvious peak shift was observed by SEC, similar to the change induced by glutathionylation, as observed previously (41). The incubated samples were then subjected to HPLC-Q-TOF MS and nanoLC-LTQ-Orbitrap XL MS/MS to identify the location of the PES modification.

**Figure 2:**
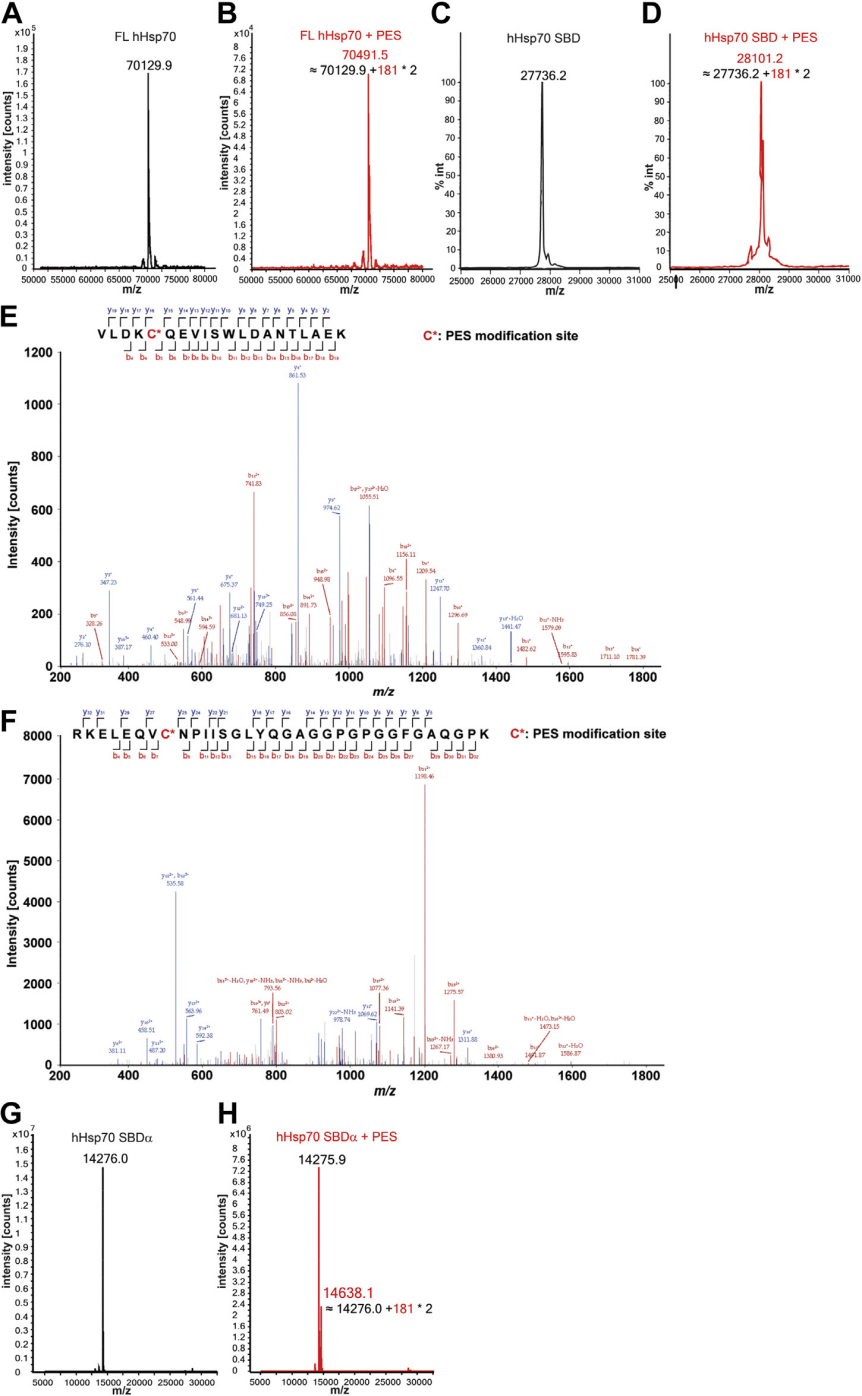
**Covalent binding of PES to hHsp70 detected by mass spectrometry.***A*–*D*, the molecular weight of full-length hHsp70 (*A*–*B*) and the SBD of hHsp70 (*C*–*D*) were detected by Q-TOF mass spectrometry before (*A* and *C*) and after (*B* and *D*) 24-h incubation with PES. After incubation with PES, both the molecular weight of full-length hHsp70 and the SBD of hHsp70 increased by ∼362 Da, which is the molecular weight of two PES molecules (181 Da). *E*–*F*, detection by mass spectrometry of PES modification at Cys-574 (*E*) and Cys-603 (*F*) of WT hHsp70 treated with PES in the presence of ADP. NanoLC-LTQ-Orbitrap XL analysis confirmed the presence of the PES-modified peptide VLDKC∗(574)QEVISWLDANTLAEK (*E*) and the PES-modified peptide RKELEQVC∗(603)NPIISGLYQGAGGPGPGGFGAQGPK (*F*) after trypsin digestion. The detected peaks (main panel) correspond to the predicted peptides (*inset*), where *red* corresponds to observed N-terminal peptide fragments and *blue* corresponds to observed C-terminal peptide fragments. C∗ indicates Cys-574 and Cys-603, which undergo PES modification. *G*–*H*, the molecular weight of the SBDα of hHsp70 was detected by Q-TOF mass spectrometry before (*G*) and after (*H*) 24-h incubation with PES. After incubation with PES, the molecular weight of most hHsp70 SBDα is unchanged, and only a small proportion shows an increased molecular weight. hHsp70, human HspA1A.

After incubation with PES, the molecular weight of full-length hHsp70 or hHsp70 SBD(385–641) increased by 362.42 Da or 365.0 Da, respectively (Fig. 2, *A*–*D*), while the molecular weight of hHsp70 NBD(1–385) did not increase, as indicated by HPLC-Q-TOF MS. These results indicate that two PES molecules interact with the SBD of each hHsp70 molecule (molecular weight of PES is 181.21 Da). NanoLC-LTQ-Orbitrap XL MS/MS was then performed on the PES-bound full-length hHsp70 to identify the modification site. The results confirmed that Cys-574 and Cys-603 undergo PES modification in ADP-bound WT hHsp70 (Fig. 2, *E*–*F*), suggesting that PES covalently reacts with Cys-574 and Cys-603 in the SBDα of hHsp70 by Michael addition (Fig. 1). However, for the isolated hHsp70 SBDα(511–641), under the same PES incubation conditions, only a very small proportion of SBDα was modified by PES (Fig. 2, *G*–*H*), suggesting that PES modification occurs more readily in full-length hHsp70 or the isolated SBD than in the further truncated SBDα alone.

### Covalent attachment of PES to hHsp70 alters the structure and function of hHsp70

**Figure 3:**
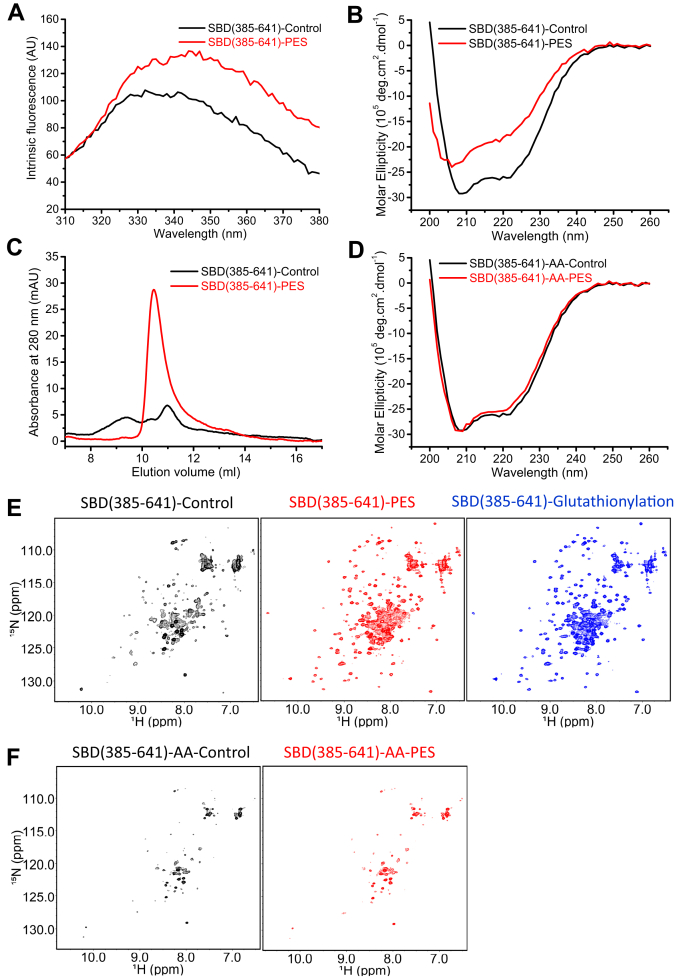
**Covalent attachment of PES to hHsp70 results in structural changes in the SBD similar to those induced by glutathionylation of Cys residues.** Conformation and secondary structure of untreated control (*black*) and PES-treated (*red*) SBD(385–641) were compared by SEC (*A*), intrinsic tryptophan fluorescence (after excitation at 295 nm) (*B*) and far-UV CD (*C*). *D*, far-UV CD spectra of control (*black*) and PES-treated (*red*) SBD(385–641)-AA were compared. *E*, ^1^H-^15^N HSQC spectra of untreated control (*black*), PES-treated (*red*), and glutathionylated (-G, *blue*) SBD(385–641) were compared. *F*, ^1^H-^15^N HSQC spectra of untreated control (*black*) and PES-treated (*red*) SBD(385–641)-AA were compared. hHsp70, human HspA1A.

In order to investigate the conformational changes that occur upon PES modification of the two C-terminal Cys residues, we used the truncation mutant hHsp70 SBD(385–641), which contains both the SBDβ substrate binding site and the SBDα lid (Table 1). The tryptophan fluorescence spectra (Fig. 3*A*) and far-UV CD spectra (Fig. 3*B*) showed significant structural change indicating loss of α-helical structure upon PES modification of the SBD. This is similar to the changes observed for glutathionylation of the SBD (41). Analytical SEC showed disappearance of oligomeric elution peaks, indicating a reduction in the degree of oligomerization of the SBD upon PES modification (Fig. 3*C*), again similar to the effects of glutathionylation (41). The higher UV absorbance of PES-modified SBD was from the absorbance of PES (Fig. 3*C*). The ^1^H-^15^N NMR HSQC spectrum of the hHsp70 SBD showed a number of weak peaks, caused by a distribution of oligomeric states (Fig. 3*E*). Upon PES modification, the ^1^H-^15^ N NMR HSQC spectrum of the hHsp70 SBD showed stronger peaks and appearance of new peaks, similar to the effects of glutathionylation (Fig. 3*E*). The changes in the NMR spectrum also suggest a transition from oligomer to monomer consistent with the analytical SEC results (Fig. 3*C*).

To test whether the structural changes are caused by cysteine modification, we incubated the C574A/C603A mutant SBD(385–641)-AA with PES. Both the far-UV CD spectra and the ^1^H-^15^N NMR HSQC spectra indicated that SBD(385-641)-AA does not undergo any changes in structure after incubation with PES (Fig. 3, *D* and *F*), which excludes an effect of noncovalent binding of PES on the structure of the SBD.

**Figure 4:**
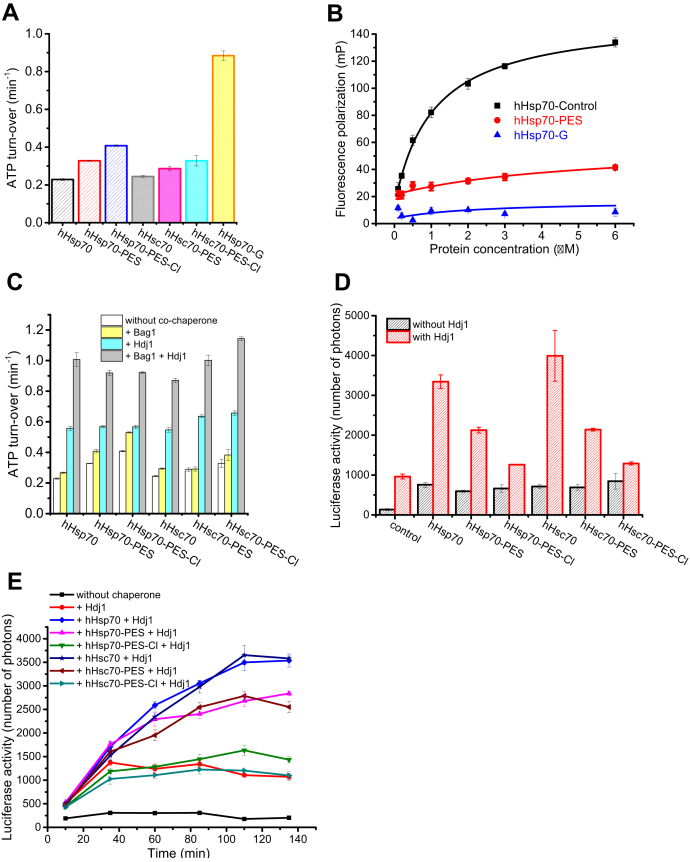
**Covalent attachment of PES to hHsp70 results in functional changes of hHsp70.***A*, the effect of PES and PES-Cl on the ATPase activity of hHsp70 and hHsc70 was detected and compared with glutathionylated hHsp70. *B*, peptide binding ability of untreated control (*black*), PES-treated (*red*), and glutathionylated (-G, *blue*) full-length hHsp70 (residues 1–641) in the presence of 0.5 mM ADP in Buffer B was compared. Fluorescence polarization (FP) at 520 nm after excitation at 485 nm was used to monitor the binding of 20 nM FITC-labeled ALLLSAPRR (FAR) peptide to different concentrations of hHsp70 or its mutants, as indicated. *C*, the stimulatory effects of cochaperones Hdj1 (2 μM) and Bag1 (0.5 μM) on ATPase activity of PES and PES-Cl modified hHsp70 and hHsc70 (1 μM) were compared. *D*–*E*, the effects of PES and PES-Cl on luciferase refolding activity of hHsp70 and hHsc70 were measured. The stimulatory effects of the cochaperone Hdj1 (0.5 μM) on luciferase refolding activity of PES and PES-Cl modified hHsp70 and hHsc70 (1 μM) were measured after 2-h refolding at 37 °C (*D*), and the time course of luciferase refolding in the presence of Hdj1 (0.5 μM) and PES and PES-Cl-modified or unmodified hHsp70 and hHsc70 (1 μM) was also compared as indicated (*E*). PES and PES-Cl-treated hHsp70 and hHsc70 were prepared by incubating hHsp70 or hHsc70 in the presence of 1 mM PES or PES-Cl and 1 mM ADP at 37 °C for 24 h followed by dialysis at 4 °C to remove PES, PES-Cl, and ADP. The data shown are the mean of three individual experiments and the error bars represent the standard error of the mean. hHSc70, human HspA8; hHsp70, human HspA1A.

The similarity in ^1^H-^15^N NMR HSQC spectra of the hHsp70 SBD upon PES modification and glutathionylation indicates similar 3-D structures of PES-modified and glutathionylated hHsp70 SBD. We previously solved the NMR structure of the glutathionylated hHsp70 SBD, which showed that glutathionylation within the SBDα leads to unfolding of the SBDα, and the unfolded C-terminal region blocks the substrate binding site through binding of residue Leu542 in the hydrophobic site of the SBDβ (41). This structure can also explain the changes in tryptophan fluorescence, far-UV CD, and SEC profile of PES-modified SBD. It is likely that a covalent inhibitor of hHsp70 could have similar effects as glutathionylation to turn off chaperone activity of hHsp70. Therefore, we further investigated the effect of PES modification on function of hHsp70 by ATPase assay, peptide binding assay, and luciferase refolding assay. The PES-modified hHsp70 had increased ATPase activity and decreased peptide binding ability, similar to glutathionylated hHsp70 (Fig. 4, *A*–*B*). However, the amplitude of increase in ATPase activity of PES-modified hHsp70 was smaller than for glutathionylated hHsp70 (Fig. 4*A*). PES-Cl is a derivative of PES and shows higher efficiency in killing cancer cells than PES (25). Interaction of PES-Cl with Hsp70 has also been reported (25). We compared the ATPase activity of hHsp70 and hHsc70 after treatment with PES or PES-Cl and found that the ATPase activity was higher after PES-Cl treatment than PES treatment, but still not as high as glutathionylation (Fig. 4*A*). The acceleratory effects of the cochaperones Hdj1 and Bag1 on ATPase activity of hHsp70 and hHsc70 were similar or only slightly increased after PES or PES-Cl treatment (Fig. 4*C*). Luciferase refolding activity of hHsp70 and hHsc70 was enhanced two- to three-fold by Hdj1 (Fig. 4, *D*–*E*). PES or PES-Cl treatment weakened the promotion effect of Hdj1 on luciferase refolding activity of hHsp70 and hHsc70, with PES-Cl treatment causing greater disruption of this cooperation with Hdj1 (Fig. 4, *D*–*E*). The smaller effects of PES or PES-Cl modification compared with glutathionylation on the function of hHsp70 and hHsc70 may reflect a more modest structural perturbation by PES and PES-Cl, or may reflect a lower proportion of modified protein after PES or PES-Cl treatment compared with glutathionylation. The greater effect of PES-Cl treatment on hHsp70 and hHsc70 function than PES is consistent with the higher efficiency of PES-Cl than PES in killing cancer cells.

### Covalent attachment of PES to the SBDα of hHsp70 is affected by subtle conformational adjustments in the SBDα

**Figure 5:**
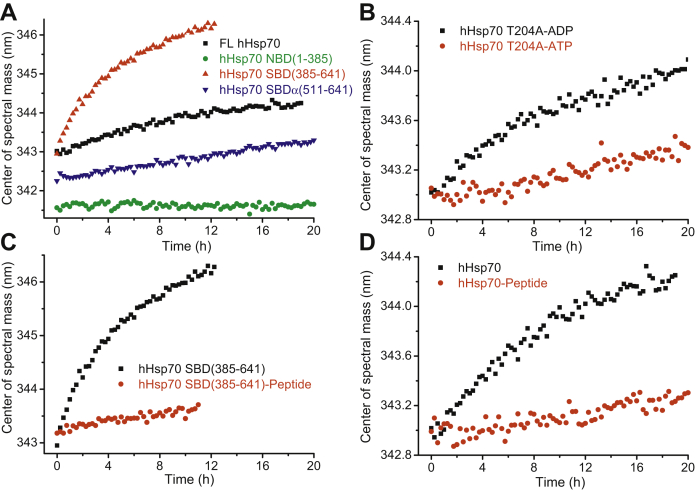
**Covalent binding of PES to the SBDα of hHsp70 is affected by domain communication and allostery of hHsp70.** The time course of conformational changes accompanying PES modification of 10 μM hHsp70 or its mutants was recorded by monitoring the CSM of the intrinsic fluorescence spectrum. PES modification was induced by 1 mM PES. *A*, PES modification kinetics of full-length hHsp70, hHsp70 NBD(1–385), hHsp70 SBD(385–641), and hHsp70 SBDα(511–641) in the presence of 0.5 mM ADP were compared. *B*, PES modification kinetics of hHsp70 T204 in the presence of 0.5 mM ADP or ATP were compared. *C*, PES modification kinetics of hHsp70 SBD(385–641) in the absence or presence of 1 mM AR peptide (ALLLSAPRR) were compared. *D*, PES modification kinetics of full-length hHsp70 in the presence of 0.5 mM ADP and in the absence or presence of 1 mM AR peptide were compared. hHsp70, human HspA1A.

The weaker reactivity of PES with the isolated SBDα than with full-length hHsp70 or the complete SBD raises questions about the effect of interdomain communication and allostery on susceptibility to undergo PES modification. We previously measured cysteine modification kinetics by monitoring changes in CSM (center of spectral mass) (41) and found that cysteine modification in the SBDα causes the peak of the intrinsic fluorescence spectrum to undergo a red shift, reflecting unfolding of the SBDα, and thus a change in the environment of the tryptophan residue that is located within the SBDα (Trp-580 in both hHsp70 and hHsc70). There is an additional Trp residue in the NBD of hHsp70 (Trp-90 in hHsp70). Here, we first measured the CSM shift for the isolated NBD in the presence of ADP and PES and observed no change after 20 h (Fig. 5*A*), suggesting there is no interaction of PES with the NBD. We then compared the PES interaction kinetics for SBDα(511–641), SBD(385–641), and full-length hHsp70 by monitoring the time course of changes in CSM when incubating hHsp70 with PES (Fig. 5*A*). From the curve (Fig. 5*A*) we calculated the half time of the reaction (*t*_1/2_) for PES modification of Cys-574 and Cys-603 under different conditions: the *t*_1/2_ for hHsp70 is 4.82 ± 0.21 h, the *t*_1/2_ for SBD(385–641) is 2.45 ± 0.10 h, and the *t*_1/2_ for SBDα(511–641) is too long to be detected (Table 2). This suggests that interaction between the SBDα and SBDβ facilitates PES reaction with the SBDα, and interaction between the NBD and SBD hinders PES modification.

**Table 1:** Summary of human HspA1A (hHsp70) and HspA8 (hHsc70) mutants used in this study

Name of protein	Description
WT hHsp70	hHsp70 (HspA1A/B)
hHsp70 NBD(1–385)	hHsp70 Δ386–641, NBD of hHsp70
hHsp70 SBD(385–641)	hHsp70 Δ1–384, SBD of hHsp70
hHsp70 SBD(385–641)-AA	hHsp70 Δ1–384/C574A/C603A
hHsp70 SBDα(537–610)	hHsp70 Δ1–536/Δ611–641, SBDα of hHsp70 consisting of the remote part of α-helix B and α-helices C and D
hHsp70 SBDα(511–641)	hHsp70 Δ1–510, including the intact SBDα of hHsp70 consisting of α-helices A–D and the intact C terminal random coil region
hHsp70 SBDα(525–641)	hHsp70 Δ1–524, including SBDα of hHsp70 consisting of α-helices B–D and the intact C terminal random coil region
hHsp70 SBDα(537–641)	hHsp70 Δ1–524, including SBDα of hHsp70 consisting of the remote part of α-helix B, α-helices C and D, and the intact C terminal random coil region
hHsp70 T204A	hHsp70 mutant, which binds but does not hydrolyze ATP
hHsp70-CSSCA	hHsp70 C267S/C306S/C603A
hHsp70-CSSAC	hHsp70 C267S/C306S/C574A
WT hHsc70	hHsc70 (HspA8)
hHsc70 SBD(385–646)	hHsc70 Δ1–384, SBD of hHsp70
hHsp70-hHsc70(α)	Chimera containing hHsp70 Δ511–641 and hHsc70 Δ1–510

hHsc70, human HspA8; hHsp70, human HspA1A.

**Table 2:** Half time of reaction (*t*_1/2_) for PES modification of Cys-574 and Cys-603 under different conditions

Protein state	*t*_1/2_ (h)
hHsp70-ADP	4.82 ± 0.21
hHsp70-Peptide	ND
hHsp70 SBD(385–641)	2.45 ± 0.10
hHsp70 SBD(385–641)-Peptide	ND
hHsp70 NBD(1–385)-ADP	ND
hHsp70 T204A-ADP	5.23 ± 0.31
hHsp70 T204A-ATP	ND
hHsc70-ADP	19.30 ± 1.96
hHsp70-hHsc70(a)-ADP	8.56 ± 0.30
hHsc70 SBD(386–646)	4.99 ± 0.19
hHsp70 SBDα(511–641)	ND
hHsp70 SBDα(537–610)	4.71 ± 0.10

hHsc70, human HspA8; hHsp70, human HspA1A.

ATP, ADP, and peptide substrate can affect interaction between the NBD and SBD and cause allosteric conformational changes in Hsp70 (12). Therefore we investigated the effect of ATP, ADP, and peptide on PES modification kinetics of hHsp70 and its mutants. hHsp70 T204A has extremely weak ATP hydrolysis ability, thus it was used to detect the effect of nucleotide binding. In the presence of ATP, the reaction rate of hHsp70 T204A was much slower than in the presence of ADP (Fig. 5*B* and Table 2). The *t*_1/2_ for hHsp70 T204A-ADP (5.23 ± 0.31 h) is similar to the *t*_1/2_ for hHsp70-ADP, whereas the *t*_1/2_ for hHsp70 T204A-ATP is too long to be detected, as for SBDα(511–641). In the presence of AR peptide, the reaction rate of both SBD(385–641) and full-length hHsp70 became much slower, and their *t*_1/2_ values were too long to be detected (Fig. 5, *C*–*D* and Table 2). Both ATP binding and peptide binding may be expected to affect interaction between the SBDα and SBDβ. ATP binding leads to detachment of the SBDα from the SBDβ and causes the SBDα to lean on the NBD. This indicates that the interaction between the SBDβ and SBDα facilitates PES modification of the SBDα, but the interaction between the NBD and SBDα does not. Although the SBDα still covers the SBDβ when peptide is bound to the SBD, the conformation of the SBDα may be subtly affected by the peptide directly or indirectly. Interaction between the SBDβ and SBDα is probably affected by peptide binding to the SBD, or there is some interaction between the peptide and the SBDα, leading to a change in PES reactivity.

**Figure 6:**
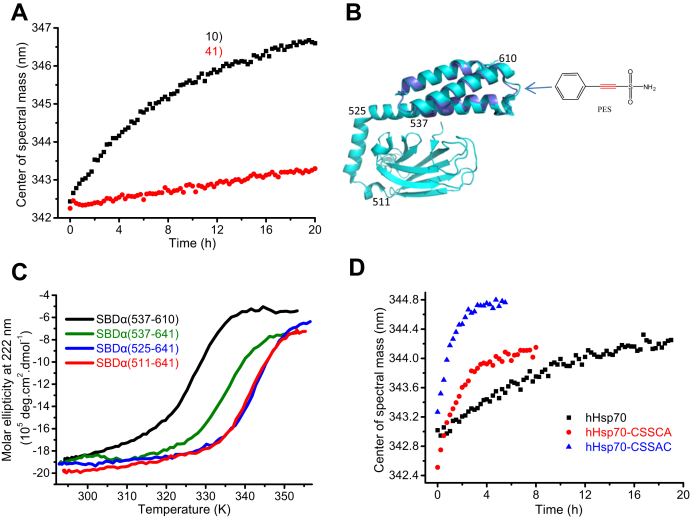
**Covalent attachment of PES to the SBDα of hHsp70 is affected by the conformational dynamics of the SBDα.** Measurement of PES modification kinetics was performed as in Figure 5. *A*, PES modification kinetics of hHsp70 SBDα(537–610) and hHsp70 SBDα(511–641) were compared. *B*, PES modification kinetics of full-length hHsp70, hHsp70-CSSCA and hHsp70-CSSAC in the presence of 0.5 mM ADP were compared. *C*, the crystal structure of the SBD of hHsp70 (PDB code 4PO2, in *blue*) and the NMR structure of the isolated SBDα(537–610) of hHs70 (PDB code 2LMG, in *violet*) were aligned. The *arrow* indicates the possible PES binding site in hHsp70. *D*, thermal denaturation of hHsp70 SBDα(537–610), SBDα(537–641), SBDα(525–641), and SBDα(511–641) was monitored by the CD signal at 222 nm. hHsp70, human HspA1A.

Regarding the high dependence on the presence of SBDβ for PES modification of SBDα, we initially suspected that PES might first bind to the SBDβ and then undergo covalent bonding to the SBDα. However, we failed to obtain crystals of the noncovalent complex between PES and the C575A/C603A mutants of the SBD or SBDα to determine the binding site of PES. Unexpectedly, we found that covalent attachment of PES occurs much more rapidly for SBDα(537–610) than SBDα(511–641) (Fig. 6*A* and Table 2), suggesting that interaction of PES with the SBDα does not require the SBDβ *per se*, but that the conformation or dynamics of the SBDα are crucial. Structural comparison indicates that the structure of the truncated SBDα(537–610) is essentially the same as the SBDα in the context of the complete SBD (Fig. 6*B*), but SBDα(537–610) is less stable as indicated by thermodynamic stability data (Fig. 6*C*). We found previously that the mutations C574A and C603A can affect the conformational dynamics of the SBDα of hHsp70, and reduced stability or increased dynamics increases susceptibility to undergo modification (41). Here we observed a marked increase in the PES reaction rate for hHsp70-CAACA and hHsp70-CAAAC compared with WT hHsp70 (Fig. 6*D*), confirming the importance of the conformational dynamics of the SBDα for PES covalent modification, and that the conformational dynamics of the SBDα is readily perturbed by interactions with the SBDβ or substrate as well as by mutation.

**Figure 7:**
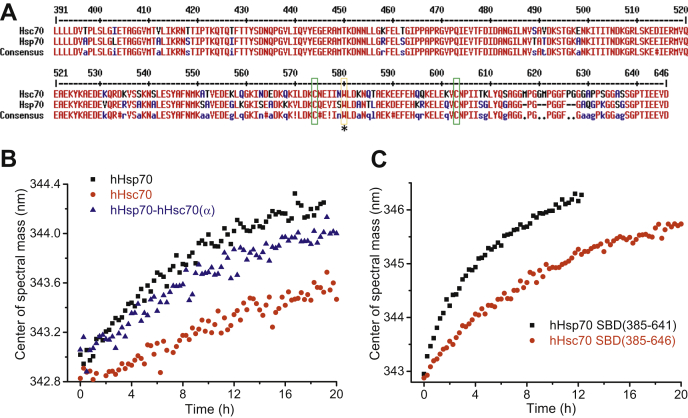
**Difference between hHsp70 and hHsc70 in covalent binding of PES.** Measurement of PES modification kinetics was performed as in Figure 5. *A*, alignment of the SBDs of hHsp70 and hHsc70, with Cys-574 and Cys-603 indicated by *boxes*, and Trp-580 indicated with an *asterisk*. *B*, PES modification kinetics of hHsp70, hHsc70, and the chimeric hHsp70-hHsc70(α) (see Table 1) in the presence of 0.5 mM ADP were compared. *C*, PES modification kinetics of hHsp70 SBD(385–641) and hHsc70 SBD(385–646) were compared. hHsc70, human HspA8.

hHsc70 was also identified as a target of PES by some researchers, but with weaker effects than for hHsp70 (24, 42). hHsc70 and hHsp70 share 85% identity, but the hHsp70 SBDα(511–641) and hHsc70 SBDα(511–646) share only 71% identity (Fig. 7*A*). The SBDs of both hHsp70 and hHsc70 contain the conserved residues Cys-574, Cys-603, and Trp-580 (Fig. 7*A*). We compared the PES reaction kinetics for hHsp70 and hHsc70 and found both full-length hHsc70 and hHsc70 SBD have much lower reaction rates than full-length hHsp70 and hHsp70 SBD (Fig. 7, *B*–*C* and Table 2). Similar to hHsp70, full-length hHsc70 also reacts more slowly with PES than the hHsc70 SBD (Fig. 7, *B*–*C* and Table 2). To test whether the SBDα or SBDβ of hHsp70 or hHsc70 determines the PES reaction rate, we monitored the reaction kinetics of a chimera composed of the NBD and SBDβ from hHsp70 and the SBDα from hHsc70. Interestingly, we found that this chimera has an intermediate PES reaction rate compared with WT hHsp70 and hHsc70 (Fig. 7*B* and Table 2), highlighting again the importance of the interaction between the SBDα and the SBDβ for PES modification of the SBDα and also indicating that the difference in PES reactivity of hHsp70 and hHsc70 depends on both the conformational dynamics of the SBDα and interaction between the SBDα and SBDβ.

**Figure 8:**
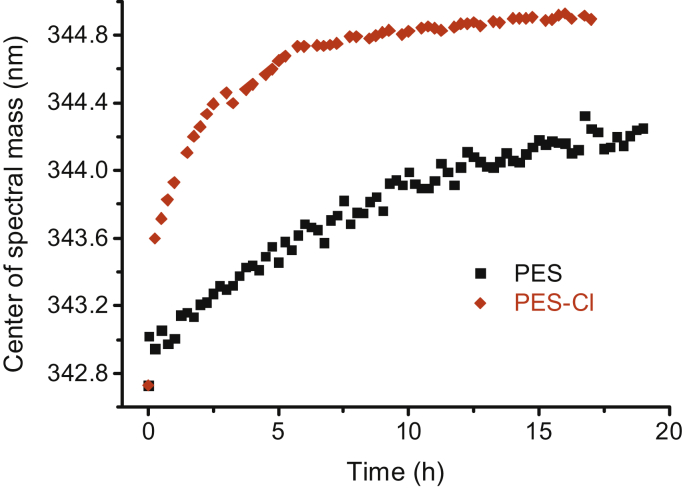
**Difference between PES and PES-Cl in covalent binding to hHsp70.** Measurement of modification kinetics was performed as in Figure 5, in the presence of 0.5 mM ADP. hHsp70, human HspA1A.

We found that interaction of the PES derivative PES-Cl and Hsp70 also results in a red shift of the peak in the intrinsic fluorescence spectrum, similar to PES modification and glutathionylation (Fig. 8). We compared the reaction kinetics of PES-Cl and PES for interaction with hHsp70 and found that the *t*_1/2_ for hHsp70 and PES (4.52 ± 0.29 h) is nearly four times longer than the *t*_1/2_ for hHsp70 and PES-Cl (1.00 ± 0.07 h) (Fig. 8 and Table 2). Therefore, the higher efficiency of PES-Cl in killing cancer cells correlates with its higher reactivity with hHsp70 and stronger effect on function of hHsp70 (Fig. 4, *A*, *D* and *E*), strongly suggesting that cytotoxicity of PES and PES-Cl toward cancer cells is related to their ability to covalently target hHsp70.

## Discussion

Due to its role in some types of cancers, Hsp70 is the next most promising molecular chaperone drug target after Hsp90 (21). PES, which was first identified in screens for inhibitors of p53, was later described as an Hsp70 inhibitor and shows high cytotoxicity in some tumor cells (24, 31). However, the mechanism of selective lethality of PES toward cancer cells and direct inhibition of hHsp70 remained unclear. In this study we detected covalent binding of PES to hHsp70 through Cys-574 and Cys-603 in the SBDα, which explains the previous reports of interaction between hHsp70 and PES. The similar effects of PES modification and glutathionylation of the SBDα on the structure and function of hHsp70 suggest that PES could have a similar effect on hHsp70 by causing unfolding of the SBDα; these results provide insight for development of new inhibitors of Hsp70. Subtle conformational regulation of the SBDα was found to affect the efficiency of PES interaction with Hsp70, meaning that PES could be used as a probe for exploring allosteric conformational changes of hHsp70 and subtle conformational differences between hHsp70 and hHsc70.

Most studies of the interaction between Hsp70 and PES were performed by George and coworkers (24, 25, 36). Our findings are consistent with their results. Using *in silico* methods and mutational analysis, George and coworkers were convinced that PES could bind to hHsp70 through its SBDα, although confirming the presence of a direct interaction had remained elusive (16, 24, 36). From this study, we can highlight some important aspects of PES covalent interaction with hHsp70: i) Sufficient interaction time is needed for the covalent reaction to proceed to completion, since PES is not a particularly reactive Michael acceptor. When PES was added into cultured cells or purified protein, a 24-h incubation time was sufficient to detect covalent interaction of PES with hHsp70. For PES-Cl the incubation time could be reduced since it has a higher reaction rate with hHsp70. ii) A high ratio of PES to hHsp70 increases the proportion of covalently modified hHsp70. Similarly, George and coworkers found that injecting hHsp70 into PES (not injecting PES into hHsp70) allowed the interaction between them to be detected by ITC (36). It is not clear whether the interaction detected by ITC involves covalent binding. It is possible that the interaction between PES and hHsp70 could include two steps: initial weak non-covalent binding followed by irreversible covalent reaction. iii) Non-ATP and nonsubstrate binding states of hHsp70 are more susceptible to covalent reaction with PES. George and coworkers have also reported that ATP and peptide substrate disfavor the interaction between PES and hHsp70 (36). In this study we found that interaction between the SBDα and SBDβ significantly affects the susceptibility of Hsp70 to undergo PES covalent modification, which explains how ATP and substrate lessen PES reactivity by inducing allosteric conformational changes throughout the entire hHsp70 molecule.

Another PES derivative, PET-16, which contains the same phenyl group and alkynyl moiety as PES, competes with PES for binding to hHsp70; the crystal structure of the complex of PET-16 and the DnaK SBD as well as mutational studies indicate that PET-16 may also bind to the same SBDβ loop region of hHsp70 (36). The covalent interaction between PES and the SBDα of hHsp70 suggests that PET-16 may also undergo covalent interaction with the SBDα. Thus there are two likely mechanisms for the competition between PES and PET-16 for binding to hHsp70: 1) if both of them can bind to the SBDα of hHsp70, then they compete for covalent reaction with cysteines in the SBDα; 2) if they bind to different sites of hHsp70, then prebinding of one compound changes the allostery and domain interactions of hHsp70, then further alters the binding ability of the other compound, since interaction between PES and hHsp70 is sensitive to allosteric conformational changes in hHsp70, as suggested in this study, and binding of PET-16 to Hsp70 is also related to allostery of Hsp70, as suggested in the previous study (36).

In this study we found that PES is very sensitive to fine conformational changes in the SBDα, indicating that the conformation of the SBDα is sensitive to its microenvironment, and this could play a role in functional regulation of hHsp70. Consistent with our previous work on glutathionylation of hHsp70 (41), this study shows that posttranslational modification within the SBDα can turn off hHsp70 function by altering interaction between the SBDα and SBDβ. Thus the SBDα is not only a lid to cover the SBDβ and stabilize substrate binding, but also a functional regulatory module for hHsp70. Comparing the kinetics of the PES reaction for isolated hHsp70 SBD, ADP-bound full-length hHsp70, ATP-bound full-length hHsp70, and isolated hHsp70 SBDα, we found that the reaction rate sequence is hHsp70 SBD > ADP-bound hHsp70 > ATP-bound hHsp70 ≈ hHsp70 SBDα. This indicates that docking between the NBD and SBD slows the reaction by detaching the SBDα and SBDβ, and there is a significant minor fraction of the docked conformation in ADP-bound hHsp70. For hHsc70 we can also infer that a significant fraction of docked structure exists in its ADP-bound state. This is consistent with recent research regarding the heterogeneous nature of hHsp70 and hHsc70 (13, 14).

Another covalent inhibitor of hHsp70, necroptosis-blocking compound 1 (NBC1), was recently reported to covalently interact with Cys-574 and Cys-603 (43). NBC1 and PES-Cl show a similar inhibition effect on mixed lineage kinase domain-like protein (MLKL) polymerization and necroptosis by targeting hHsp70 (43). The same study also implies involvement of Cys-574 and Cys-603 in MLKL polymerization (43). NBC1 may have a similar effect on the structure and function of hHsp70 as detected for PES in this study. Novolactone, which can covalently react with Glu-444 of hHsp70, also shows a promising inhibition effect on function of hHsp70 (27). Development of covalent inhibitors targeting cysteine residues in kinases has been a focus for cancer therapy for a long time, as the irreversibility of covalent binding prolongs pharmacodynamics, and there is potential for flexible rational design to give high potency and high specificity (44, 45, 46). In addition to acrylamide inhibitors, alkyne inhibitors targeting thiols in proteins also show promise for drug development (44, 47). hHsp70, which contains five cysteine residues, is another potential target for cancer therapy. While the development of covalent inhibitors targeting cysteines is still at an initial stage, inhibitors that have been identified include YK5 targeting Cys-267 (23), methylene blue targeting Cys-306 (48), and NBC1 (43), PES and PES-Cl targeting Cys-574 and Cys-603. The development of covalent inhibitors of hHsp70 targeting Cys-574 and Cys-603 is a promising strategy for drug design. Compound libraries could be screened for compounds that can covalently react with protein thiols, as these would be worth testing as candidates for hHsp70 inhibitors. Development of PES or NBC1 derivatives or other thiol reactive compounds could be further optimized for reaction efficiency and specificity, as a step toward drug development.

Reaction with PES is sensitive to the conformational dynamics of the hHsp70 SBDα and differs between hHsp70 and hHsc70, indicating some selectivity of the PES reaction. Whether Hsp70 is the specific target of PES is still to be explored. Some studies have suggested that PES can interfere with redox in cancer cells (35, 49) and ROS levels are related to the cytotoxicity of PES (37, 50), indicating PES may attack thiols from additional targets, such as GSH. In this study, the PES reaction with hHsp70 required high concentrations of PES, much higher than the actual concentration needed to efficiently kill cancer cell lines (24). There are still questions awaiting further study, such as whether the covalent binding of PES to hHsp70 occurs in living cells at low concentrations of PES and whether there are other targets of PES contributing to the cytotoxicity of PES in addition to the inhibition of hHsp70.

## Experimental procedures

### Protein expression and purification

The human *HSPA1A* gene (51) (UniProtKB code: P0DMV8) and *HSPA8* gene (UniProtKB code: P11142), which were kindly provided by Prof. Richard Morimoto, Northwestern University, were subcloned into the pET28a-smt3 expression plasmid for expression of hHsp70 with a His6-Smt3 tag (52). The hHsp70 Cys to Ala point mutants and the hHsp70 domain-deletion mutants (Table 1) were derived from the human *HSPA1A* gene as described (41). The chimeric construct described in the text was derived from the *HSPA1A* and *HSPA8* genes by three-time PCR.

Expression and purification of hHsp70, hHsc70, and their mutants were performed as described (41, 53). All protein concentrations are given in terms of monomer and were determined using a bicinchoninic acid (BCA) assay kit (Pierce).

### Preparation of PES-modified hHsp70 and hHsc70

To prepare PES-modified hHsp70, 10 μM of hHsp70 (or its mutant) was mixed with 1 mM PES (Sigma Aldrich, dissolved in DMSO) and allowed to stand in the dark at 37 °C for 24 h in order to allow PES modification. Unbound PES was then removed by dialysis. For full-length hHsp70, ADP (final concentration 1 mM) was added to the protein before PES was added, while for hHsp70 mutants lacking the NBD, ADP was not added. The method for preparation of PES-modified hHsc70 was the same.

### Mass spectra detection of PES modification of hHsp70

Q-TOF MS and nanoLC-LTQ-Orbitrap XL MS/MS were performed to detect PES modification of hHsp70. Control and PES-treated WT hHsp70 (4 μl) or hHsp70 mutants (18 μl) were loaded onto the Q-TOF MS instrument after separation by HPLC. Profile spectra of 600 to 1800 M/Z were collected and deconvoluted using Deconvolute (MS): protein software. The deconvoluting algorithm is Maximum Entropy and the scale of molecular weight is 5000 to 80,000 Da.

For nanoLC-LTQ-Orbitrap XL MS/MS, trypsin-digested peptides were separated by C18 reverse-phase column (filled with 3 μm ReproSil-Pur C18-AQ from Dr Maisch GmbH, Ammerbuch) and loaded using a C18 reverse-phase column (filled with 5 μm ReproSil-Pur C18-AQ from Dr Maisch GmbH, Ammerbuch) onto LTQ-Orbitrap MS/MS. Data were analyzed as described by Proteome Discoverer software (version 1.4.0.288, Thermo Fischer Scientific) (41). The second MS spectra were searched in the human database (uniprot_human_proteome_20160229_con) using the SEQUEST search engine. PES modification of cysteine and oxidation of methionine were set as variable modifications. The matching of searched peptide and MS spectra (PSM) was filtered by Percolator calculation.

### Intrinsic fluorescence

Intrinsic fluorescence measurements were carried out on a Hitachi F-4500 instrument. The intrinsic florescence spectra of control and PES-modified hHsp70 or its mutants were measured between 310 and 400 nm, using excitation wavelengths of 295 nm at 25 °C. The proteins were prepared in Buffer A (50 mM Tris-HCl buffer, pH 7.5, containing 100 mM KCl and 5 mM MgCl_2_). The purity of ATP (sigma A2383 ≥99%) and ADP (Sigma A2754 ≥95%) used in this study was checked by HPLC; ATP showed no detectible impurities (>99% purity), whereas ADP is generally >92% pure (including 1.2% ATP and 6.4% AMP).

To monitor the shift of center of spectral mass (CSM) for the intrinsic fluorescence spectra caused by PES modification of hHsp70, 1 mM PES was rapidly mixed with 10 μM WT-hHsp70, WT-hHsc70, or their mutants in Buffer A in the presence of 0.5 mM ADP or ATP before the spectra were recorded at 37 °C every 30 s (for fast reactions) or 10 min (for slow reactions) until the intrinsic fluorescence signal reached a plateau; spectra were recorded between 310 nm and 380 nm with excitation at 295 nm. For hHsp70 mutants lacking the NBD, nucleotide was not added. AR peptide (ALLLSAPRR) at a concentration of 1 mM was added as needed. The CSM of intrinsic fluorescence was calculated using the following formula:

$$CSM\;=\;\frac{\sum_{i=310}^{380}i\;\times \;IF_{i}}{\sum_{i=310}^{380}IF_{i}}$$

The plots of the CSM value (y axis) *versus* reaction time (x axis) were fitted using the following formula to calculate the half time of the reaction (*t*_1/2_) for PES modification:

$$\text{ y}\;={\text{ y}}_{0}\;+\text{ A}\;\times \;{\text{e}}^{\frac{\left(-\text{x}\right)\;\times \;\ln\;2}{{\text{t}}_{1/2}}}$$

### Circular dichroism

Far-UV circular dichroism (CD) spectra were measured between 200 and 250 nm on a Chirascan Plus CD instrument (Applied Photophysics, UK) at 25 °C in a 1 mm path-length thermostated cuvette after preincubation for 10 min at 25 °C. Spectra of control or PES-treated hHsp70 or its mutants were compared in Buffer A.

Temperature-induced denaturation measurements were performed under the following conditions: 5 μM hHsp70 truncation mutants were prepared in Buffer A. Denaturation was followed by monitoring of the increase in ellipticity at 222 nm. A temperature ramp of 0.5 °C/min was applied between 25 °C (298 K) and 95 °C (368 K). All equilibrium measurements were performed using a Chirascan Plus CD instrument (Applied Photophysics, UK) in a 1 mm path-length thermostated quartz cuvette. Data were collected with a band pass of 1 nm and the sensitivity was set to 100 mdeg.

### Size-exclusion chromatography assay

The oligomeric state of control and PES-treated hHsp70 or its mutants were compared by SEC (Superdex 200 10/300 GL column or Superdex 75 10/300 GL column, GE) in Buffer A at RT. Beta-amylase (200 kDa), alcohol dehydrogenase (150 kDa), bovine serum albumin (66 kDa), ovalbumin (45 kDa), carbonic anhydrase (29 kDa), PSMF-treated trypsinogen (24 kDa), and cytochrome c (12.4 kDa) were used as molecular mass standards.

### NMR experiments and structure calculations

^15^N-labeled hHsp70 SBD(385–641) and hHsp70 SBD(385–641)-AA were prepared using the same procedures as for WT hHsp70, except that cells were grown in M9 minimal medium containing ^15^NH_4_Cl as the sole nitrogen source. PES-treated samples were prepared as above. NMR experiments were performed on an Agilent DD2 (DirectDrive 2) 600 MHz spectrometer equipped with a cryo-probe. The ^1^H-^15^N HSQC spectra of 0.2 mM control and PES-treated hHsp70 SBD(385–641) or hHsp70 SBD(385–641)-AA were acquired in Buffer A with 10% (v/v) D_2_O. All experiments were performed at 298 K. Data were processed with NMRPipe and analyzed with NMRViewJ.

### ATPase assay (malachite green)

Colorimetric determination of inorganic phosphate produced by ATP was performed using the malachite green reagent, prepared as described (54, 55). A 10 μl volume of control/PES-modified hHsp70 (1 μM) was mixed with 10 μl of 2 mM ATP in Buffer A in a 96-well plate. If cochaperones were added, 2 μM Hdj1 and/or 0.5 μM Bag1 was used. The plate was incubated for 4 h at 37 °C. An 80 μl volume of malachite green and 10 μl of 34% sodium citrate were added sequentially. The samples were mixed thoroughly and incubated at 37 °C for 30 min before measuring the OD_620_ on a SpectraMax M3e plate reader (Molecular Devices, USA). The rate of intrinsic ATP hydrolysis was deduced by subtracting the signal from ATP in the absence of chaperone.

### Peptide binding assay

Peptide binding assays based on fluorescence polarization (FP) were performed as described previously with slight modifications (56). Steady-state FP measurements were performed at RT with 60 min incubation in Buffer A to give the binding constant (*K*_d_). Binding was assessed by incubating increasing concentrations of control and PES-modified hHsp70 with a fixed concentration (20 nM) of fluorescently-labeled substrate (FITC-ALLLSAPRR peptide, FAR) and FP values were measured. FP measurements were performed on a Fluostar microplate reader (BMG Labtech) using the FP filter set (emission 485 and excitation 520 nm). FP values are expressed in millipolarization (mP) units. All statistical analyses were performed with Origin software. Binding data were analyzed using nonlinear regression analysis (single site binding model) in Origin software.

### Luciferase refolding assay

Hsp70-assisted luciferase refolding assays were performed as described previously (57) with slight modifications. The refolding of guanidine hydrochloride (GuHCl) denatured firefly luciferase (Promega) was performed in Buffer B containing 2 mM ATP and 2.2 mM DTT at 37 °C in the presence or absence of chaperones. PES/PES-Cl modified or unmodified hHsp70/hHsc70 and Hdj1 were added into the refolding system at final concentrations of 1 μM and 0.5 μM respectively. Each reaction was performed in triplicate, and 5 μl of the refolding mixture was removed and added to a white flat-bottomed 96-well plate (JET Biofil) that was preloaded with 10 μl of SteadyGlo (Promega). After mixing, the luminescence was measured on a SpectraMax M3e multimode plate reader (Molecular Devices, USA) using a 500-ms integration time. Aliquots were removed every 20 to 30 min during incubation at 37 °C to monitor the time course of luciferase refolding. The endpoints of luciferase refolding were measured after incubation for 2 h at 37 °C.

## Data availability

All data are contained within the article.

## Conflict of interest

The authors declare that they have no conflicts of interest with the contents of this article.
